# Supplementary material for: Twist piezoelectricity: giant electromechanical coupling in magic-angle twisted bilayer LiNbO3
Source: Nat Commun. 2024 Jun 12;15:5002. doi: 10.1038/s41467-024-49321-x (PMC11169249; doi:10.1038/s41467-024-49321-x)
Supplement: Supplementary file 1 — Supplementary Information [file 41467_2024_49321_MOESM1_ESM.pdf]

Supplementary Information

Twist piezoelectricity: giant electromechanical coupling in magic-angle twisted bilayer $LiNbO_3$

Supplementary Note 1. Derivation of piezoelectric coupled acoustic waves in X-cut $LiNbO_3$ crystal

A. Details of derivation

In a piezoelectric crystal, the acoustic fields equations:

$$\nabla \cdot \mathbf{T} = \rho \frac{\partial^2 \vec{\boldsymbol{u}}}{\partial t^2} \tag{S1}$$

$$\nabla_s \vec{\boldsymbol{u}} = \mathbf{S} \tag{S2}$$

where **T** is stress tensor, $\vec{\boldsymbol{u}}$ is the particle displacement, **S** is strain tensor, and the electromagnetic fields equations:

$$\nabla \times \vec{\boldsymbol{E}} = -\frac{\partial \vec{\boldsymbol{B}}}{\partial t} \tag{S3}$$

$$\nabla \times \vec{\boldsymbol{H}} = \frac{\partial \vec{\boldsymbol{D}}}{\partial t} \tag{S4}$$

where $\vec{\boldsymbol{E}}$ is electric fields, $\vec{\boldsymbol{B}}$ is magnetic induction intensity fields, $\vec{\boldsymbol{H}}$ is magnetic strength fields and $\vec{\boldsymbol{D}}$ is electric displacement, are coupled through the piezoelectric constitutive relations, which can be given in strain-charge form:

$$\vec{\boldsymbol{D}} = \boldsymbol{\epsilon}^{\mathrm{T}} \cdot \vec{\boldsymbol{E}} + \mathbf{d}\colon \mathbf{T} \tag{S5}$$

$$\mathbf{S} = \mathbf{d} \cdot \vec{\boldsymbol{E}} + \mathbf{s}^{E}\colon \mathbf{T} \tag{S6}$$

where $\boldsymbol{\epsilon}^{\mathrm{T}}$is permittivity at zero or constant stress, **d** is piezoelectric strain constants, $\mathbf{s}^E$ is compliance constants, as well as in stress-charge form:

$$\vec{\boldsymbol{D}} = \boldsymbol{\epsilon}^{\mathrm{S}} \cdot \vec{\boldsymbol{E}} + \mathbf{e}\colon \mathbf{S} \tag{S7}$$

$$\mathbf{T} = -\mathbf{e} \cdot \vec{\boldsymbol{E}} + \mathbf{c}^{E}\colon \mathbf{S} \tag{S8}$$

where $\boldsymbol{\epsilon}^{\mathrm{S}}$ is permittivity at zero or constant strain, **e** is piezoelectric stress constants, $\mathbf{c}^E$ is elastic constants at zero or constant electric field. Combine the equations S1-S8, we obtain the piezoelectrically coupled wave equations in piezoelectric crystals as given as equation (1) and equation (2) in main text of this letter. The matrix form of equation (1) and equation (2) can be expressed as:

$$\begin{pmatrix} \frac{\partial}{\partial x} & 0 & 0 & 0 & \frac{\partial}{\partial z} & \frac{\partial}{\partial y} \\ 0 & \frac{\partial}{\partial y} & 0 & \frac{\partial}{\partial z} & 0 & \frac{\partial}{\partial x} \\ 0 & 0 & \frac{\partial}{\partial z} & \frac{\partial}{\partial y} & \frac{\partial}{\partial x} & 0 \end{pmatrix} \cdot [c^E] \cdot \begin{pmatrix} \frac{\partial}{\partial x} & 0 & 0 \\ 0 & \frac{\partial}{\partial y} & 0 \\ 0 & 0 & \frac{\partial}{\partial z} \\ 0 & \frac{\partial}{\partial z} & \frac{\partial}{\partial y} \\ \frac{\partial}{\partial z} & 0 & \frac{\partial}{\partial x} \\ \frac{\partial}{\partial y} & \frac{\partial}{\partial x} & 0 \end{pmatrix} \cdot \begin{bmatrix} u_x \\ u_y \\ u_z \end{bmatrix} - \rho \frac{\partial^2}{\partial t^2} \mathbf{I}_{3\times3} \cdot \begin{bmatrix} u_x \\ u_y \\ u_z \end{bmatrix} = \begin{pmatrix} \frac{\partial}{\partial x} & 0 & 0 & 0 & \frac{\partial}{\partial z} & \frac{\partial}{\partial y} \\ 0 & \frac{\partial}{\partial y} & 0 & \frac{\partial}{\partial z} & 0 & \frac{\partial}{\partial x} \\ 0 & 0 & \frac{\partial}{\partial z} & \frac{\partial}{\partial y} & \frac{\partial}{\partial x} & 0 \end{pmatrix} \cdot [e] \cdot \begin{bmatrix} E_x \\ E_y \\ E_z \end{bmatrix} \tag{S9}$$

and

$$\begin{pmatrix} 0 & -\frac{\partial}{\partial z} & \frac{\partial}{\partial y} \\ \frac{\partial}{\partial z} & 0 & -\frac{\partial}{\partial x} \\ -\frac{\partial}{\partial y} & \frac{\partial}{\partial x} & 0 \end{pmatrix} \cdot \begin{pmatrix} 0 & -\frac{\partial}{\partial z} & \frac{\partial}{\partial y} \\ \frac{\partial}{\partial z} & 0 & -\frac{\partial}{\partial x} \\ -\frac{\partial}{\partial y} & \frac{\partial}{\partial x} & 0 \end{pmatrix} \cdot \begin{bmatrix} E_x \\ E_y \\ E_z \end{bmatrix} + \mu_0 [\epsilon^{\mathrm{S}}] \cdot \frac{\partial^2}{\partial t^2} \mathbf{I}_{3\times3} \cdot \begin{bmatrix} E_x \\ E_y \\ E_z \end{bmatrix} = -\mu_0 \frac{\partial^2}{\partial t^2} [e]^{\mathrm{T}} \begin{pmatrix} \frac{\partial}{\partial x} & 0 & 0 \\ 0 & \frac{\partial}{\partial y} & 0 \\ 0 & 0 & \frac{\partial}{\partial z} \\ 0 & \frac{\partial}{\partial z} & \frac{\partial}{\partial y} \\ \frac{\partial}{\partial z} & 0 & \frac{\partial}{\partial x} \\ \frac{\partial}{\partial y} & \frac{\partial}{\partial x} & 0 \end{pmatrix} \begin{bmatrix} u_x \\ u_y \\ u_z \end{bmatrix} \tag{S10}$$

where $[c^E]$ is a 6×6 sized matrix, representing the elastic constants of LN and $[e]$ is a 6×3 sized matrix, representing the piezoelectric constants of LN and $[\epsilon^S]$ is a 3×3 sized matrix, representing the permittivity constants of LN:

$$[c^E]=\begin{bmatrix} c_{11} & c_{12} & c_{13} & c_{14} & 0 & 0\\ c_{12} & c_{11} & c_{13} & -c_{14} & 0 & 0\\ c_{13} & c_{13} & c_{33} & 0 & 0 & 0\\ c_{14} & -c_{14} & 0 & c_{44} & 0 & 0\\ 0 & 0 & 0 & 0 & c_{55} & c_{56}\\ 0 & 0 & 0 & 0 & c_{56} & c_{66}\end{bmatrix} \tag{S11}$$

$$[e]=\begin{bmatrix} 0 & -e_{22} & e_{31}\\ 0 & e_{22} & e_{31}\\ 0 & 0 & e_{33}\\ 0 & e_{15} & 0\\ e_{15} & 0 & 0\\ e_{16} & 0 & 0\end{bmatrix} \tag{S12}$$

$$[\epsilon^S]=\begin{bmatrix} \epsilon_{11} & 0 & 0\\ 0 & \epsilon_{11} & 0\\ 0 & 0 & \epsilon_{33}\end{bmatrix} \tag{S13}$$

Noted that c_{55} equals to c_{44}, c_{56} equals to c_{14} and c_{66} equals to $(c_{11}-c_{12})/2$ as well as e_{16} equals to $-e_{22}$. Since the solutions are plane waves propagating along X axis of $LiNbO_3$, all corresponding components of field should take form of $\exp(j\omega t-jkx)$. Thus the operators $\partial/\partial y$ and $\partial/\partial z$ leads to zero during the derivation, as well as the time derivative operator $\partial/\partial t$ leads to $j\omega$. Substitute the determined propagation direction, expressed in wave vector $\vec{k}=(k,0,0)$, the wave equations (S9) and (S10) can be derived as component forms:

$$\left\{\frac{\partial^2}{\partial x^2}\begin{bmatrix} c_{11} & 0 & 0\\ 0 & c_{66} & c_{56}\\ 0 & c_{56} & c_{55}\end{bmatrix}-\rho\frac{\partial^2}{\partial t^2}\mathbf{I}_{3\times3}\right\}\begin{bmatrix} u_x\\ u_y\\ u_z\end{bmatrix}=\frac{\partial}{\partial x}\begin{bmatrix} -e_{22}E_y+e_{31}E_z\\ e_{16}E_x\\ e_{15}E_x\end{bmatrix} \tag{S14}$$

$$\left\{\begin{bmatrix} 0 & 0 & 0\\ 0 & \frac{\partial^2}{\partial x^2} & 0\\ 0 & 0 & \frac{\partial^2}{\partial x^2}\end{bmatrix}-\mu_0\frac{\partial^2}{\partial t^2}\begin{bmatrix} \epsilon_{11} & 0 & 0\\ 0 & \epsilon_{11} & 0\\ 0 & 0 & \epsilon_{33}\end{bmatrix}\right\}\begin{bmatrix} E_x\\ E_y\\ E_z\end{bmatrix}=\mu_0\frac{\partial^3}{\partial x\partial t^2}\begin{bmatrix} e_{16}u_y+e_{15}u_z\\ -e_{22}u_x\\ e_{31}u_x\end{bmatrix} \tag{S15}$$

It is obviously that the degenerated longitudinal waves (u_x) couples with yz-inplane polarization electric fields (E_y and E_z), while the shear horizontal polarized acoustic waves (u_y and u_z) only couples with longitudinal electric fields (E_x). Solving equation (S14) and equation (S15) for the coupled fields respectively, the dispersion of first longitudinal waves can be derived as:

$$(\rho\omega^2-c_{11}k^2)(\epsilon_{11}\mu_0\omega^2-k^2)(\epsilon_{33}\mu_0\omega^2-k^2)=k^2\omega^2\mu_0[e_{22}^2(\epsilon_{33}\mu_0\omega^2-k^2)+e_{31}^2(\epsilon_{11}\mu_0\omega^2-k^2)] \tag{S16}$$

As can be seen that there are three solutions to equation (S16) while one corresponding to pure longitudinal acoustic waves and the other two correspond to the ordinary light and the unusual light in LN crystal. Thus, the acoustic modes were called quasi-longitudinal wave, which coupled with shear polarized quasi-electromagnetic waves.

The dispersion of another two SH polarized waves were solved as:

$$k^2 \begin{bmatrix} c_{66} + \frac{e_{16}^2}{\epsilon_{11}} & c_{56} + \frac{e_{15}e_{16}}{\epsilon_{11}} \\ c_{56} + \frac{e_{15}e_{16}}{\epsilon_{11}} & c_{55} + \frac{e_{15}^2}{\epsilon_{11}} \end{bmatrix} \cdot \begin{bmatrix} u_y \\ u_z \end{bmatrix} = \rho\omega^2 \begin{bmatrix} u_y \\ u_z \end{bmatrix} \tag{S17}$$

Since the forms of piezoelectric coupling apply an additional coefficient calculated by $\mathbf{e}$ and $\boldsymbol{\epsilon}$, which is mathematically different from coupling form of quasi-waves, these kinds of piezoelectric acoustic waves are called as piezoelectrically stiffened waves. The longitudinal polarized coupled electric fields indicates that the curl of the electric fields maintaining zero even in time harmonic vibration and thus there is no time harmonic vibrating magnetic fields. Furthermore, the electric displacement fields should be zero indicated by equation (S4). Therefore, the coupled longitudinal electric fields can be calculated as:

$$E_x = -\frac{1}{\epsilon_{11}}(e_{15}S_5 + e_{16}S_6) \tag{S18}$$

The irrotational electric fields allows to replace electric fields vector with a scalar potential Φ as $\vec{E} = -\nabla\Phi$, which is known as the quasi-static approximation and used primarily in FEM simulations and applications.

Another important characteristic of SH stiffened waves is its distribution of stress, derived by equation (S1) and equation (S2), can be expressed as:

$$T \cdot \hat{x} = T_1\hat{x} + T_6\hat{y} + T_5\hat{z} \tag{S19}$$

with T_1 equals to zero as well as T_2, T_3 and T_4. Thus, the corresponding stress fields exhibit the same polarization as its particle velocity/displacement polarization, with the phase on the contrary because of the partial derivatives with respect to space involves an additional factor $-jk$ to its amplitude.

B. Definition of "∇" operator

According to the variable type of the object of the operator "∇", there are three types of calculations need to be defined:

$$\nabla \cdot \rightarrow \begin{pmatrix} \frac{\partial}{\partial x} & 0 & 0 & 0 & \frac{\partial}{\partial z} & \frac{\partial}{\partial y} \\ 0 & \frac{\partial}{\partial y} & 0 & \frac{\partial}{\partial z} & 0 & \frac{\partial}{\partial x} \\ 0 & 0 & \frac{\partial}{\partial z} & \frac{\partial}{\partial y} & \frac{\partial}{\partial x} & 0 \end{pmatrix} \tag{S20}$$

where the operator acted on a symmetric second order tensor (like **S** or **T**) and the result should be a vector (like $\vec{\boldsymbol{u}}$ or $\vec{\boldsymbol{E}}$),

$$\nabla \times \rightarrow \begin{pmatrix} 0 & -\frac{\partial}{\partial z} & \frac{\partial}{\partial y} \\ \frac{\partial}{\partial z} & 0 & -\frac{\partial}{\partial x} \\ -\frac{\partial}{\partial y} & \frac{\partial}{\partial x} & 0 \end{pmatrix} \tag{S21}$$

where the operator acted on a vector and the result should also be a vector,

$$\nabla_s \rightarrow \begin{pmatrix} \frac{\partial}{\partial x} & 0 & 0 \\ 0 & \frac{\partial}{\partial y} & 0 \\ 0 & 0 & \frac{\partial}{\partial z} \\ 0 & \frac{\partial}{\partial z} & \frac{\partial}{\partial y} \\ \frac{\partial}{\partial z} & 0 & \frac{\partial}{\partial x} \\ \frac{\partial}{\partial y} & \frac{\partial}{\partial x} & 0 \end{pmatrix} \tag{S22}$$

where the operator acted on a vector and the result should be a symmetric second-order tensor.

Supplementary Note 2. Derivation of partial waves in ITBLN resonant cavity

A. Partial wave methods

The dispersion characteristics and vibrating fields distributions of acoustical resonant eigenmodes in ITBLN, acoustic fields and related boundary conditions were solved by superposition of partial waves. All possible plane wave solutions which can be also called waveguide modes should be firstly solved for sake of superimposition with amplitudes and then be determined by related boundary conditions, which take form as:

$$\vec{\boldsymbol{u}} = \overrightarrow{\boldsymbol{u'}} \tag{S23}$$

$$\mathbf{T} \cdot \hat{\boldsymbol{n}} = \mathbf{T}' \cdot \hat{\boldsymbol{n}} \tag{S24}$$

where $\hat{\boldsymbol{n}}$ is the unit vector normal to the boundary. In this letter, the used partial waves were the two SH polarized waves solved in *Supplementary I* which can be expressed as:

$$\begin{cases} A_\alpha e^{-ik_\alpha \cdot x} \\ A_\beta e^{-ik_\beta \cdot x} \end{cases} \quad \begin{cases} B_\alpha e^{+ik_\alpha \cdot x} \\ B_\beta e^{+ik_\beta \cdot x} \end{cases}$$

in upper LN layer and

$$\begin{cases} D_\alpha e^{-ik_\alpha \cdot x} \\ D_\beta e^{-ik_\beta \cdot x} \end{cases} \quad \begin{cases} C_\alpha e^{+ik_\alpha \cdot x} \\ C_\beta e^{+ik_\beta \cdot x} \end{cases}$$

in lower LN layer. The subscript α and β indicates different polarization types. The reflection and refraction coefficients at the boundary between the two piezoelectric mediums require that:

$$\begin{pmatrix} B_\alpha \\ B_\beta \\ D_\alpha \\ D_\beta \end{pmatrix} = \begin{pmatrix} \Gamma^1_{\alpha\alpha} & \Gamma^1_{\alpha\beta} & \mathrm{T}^2_{\alpha\alpha} & \mathrm{T}^2_{\alpha\beta} \\ \Gamma^1_{\beta\alpha} & \Gamma^1_{\beta\beta} & \mathrm{T}^2_{\beta\alpha} & \mathrm{T}^2_{\beta\beta} \\ \mathrm{T}^1_{\alpha\alpha} & \mathrm{T}^1_{\alpha\beta} & \Gamma^2_{\alpha\alpha} & \Gamma^2_{\alpha\beta} \\ \mathrm{T}^1_{\beta\alpha} & \mathrm{T}^1_{\beta\beta} & \Gamma^2_{\beta\alpha} & \Gamma^2_{\beta\beta} \end{pmatrix} \cdot \begin{pmatrix} A_\alpha \\ A_\beta \\ C_\alpha \\ C_\beta \end{pmatrix} \tag{S25}$$

where T is refraction coefficients and Γ is reflection coefficients, solved by the boundary conditions as equation (S23) and equation (S24). To be noted that the superscript of coefficients indicates the medium from which the incident wave propagates, more specifically says, 1 indicates the upper medium and 2 indicates the lower medium. Additionally, the boundary conditions on the upper and lower sides of cavity can be expressed as a total reflected equation:

$$\begin{pmatrix} B_\alpha e^{-ik_\alpha h_1} \\ B_\beta e^{-ik_\beta h_1} \end{pmatrix} = \mathbf{I}_{2\times 2} \cdot \begin{pmatrix} A_\alpha e^{+ik_\alpha h_1} \\ A_\beta e^{+ik_\beta h_1} \end{pmatrix} \tag{S26}$$

$$\begin{pmatrix} D_\alpha e^{-ik_\alpha h_2} \\ D_\beta e^{-ik_\beta h_2} \end{pmatrix} = \mathbf{I}_{2\times 2} \cdot \begin{pmatrix} C_\alpha e^{+ik_\alpha h_2} \\ C_\beta e^{+ik_\beta h_2} \end{pmatrix} \tag{S27}$$

where $\mathbf{I}_{2\times 2}$ is second order identity matrix. Simultaneous solving equation (S17), equation (S18) and equation (S19), the determinant of dispersion characteristic and eigenfrequency can be obtained:

$$\det\begin{pmatrix} \Gamma^1_{\alpha\alpha} - e^{+2ik_\alpha h_1} & \Gamma^1_{\alpha\beta} & \mathrm{T}^2_{\alpha\alpha} & \mathrm{T}^2_{\alpha\beta} \\ \Gamma^1_{\beta\alpha} & \Gamma^1_{\beta\beta} - e^{+2ik_\beta h_1} & \mathrm{T}^2_{\beta\alpha} & \mathrm{T}^2_{\beta\beta} \\ \mathrm{T}^1_{\alpha\alpha} & \mathrm{T}^1_{\alpha\beta} & \Gamma^2_{\alpha\alpha} - e^{+2ik_\alpha h_2} & \Gamma^2_{\alpha\beta} \\ \mathrm{T}^1_{\beta\alpha} & \mathrm{T}^1_{\beta\beta} & \Gamma^2_{\beta\alpha} & \Gamma^2_{\beta\beta} - e^{+2ik_\beta h_2} \end{pmatrix} = 0 \qquad \text{(S28)}$$

B. Symmetric distribution of stress

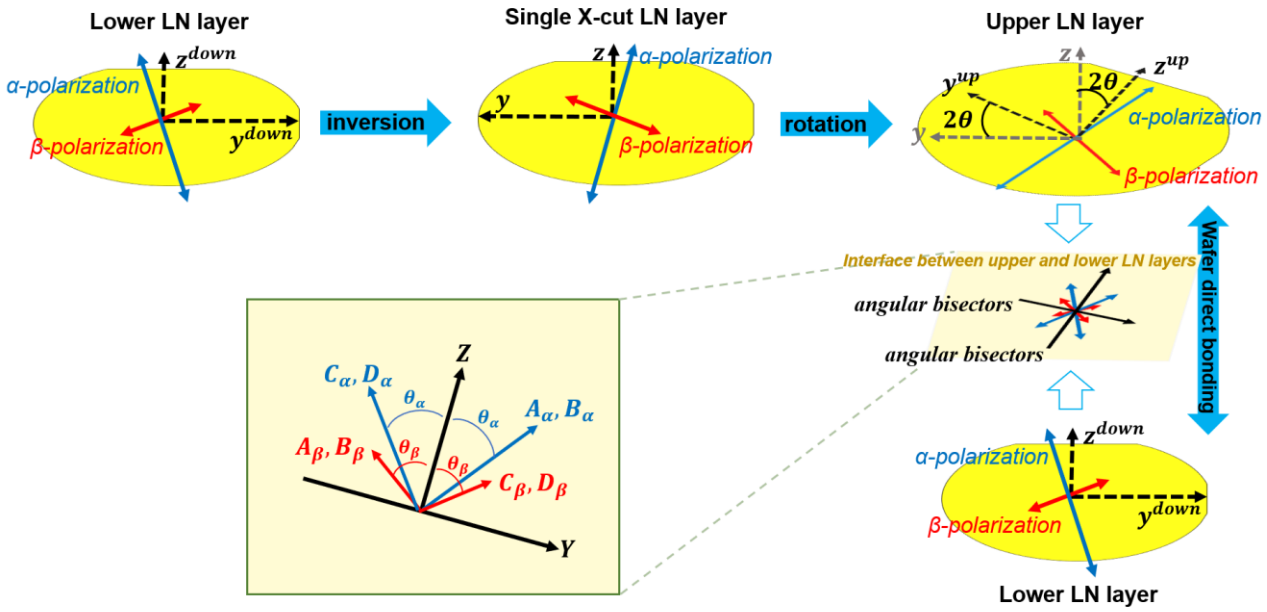

Supplementary Figure 1. Schematics of inversion and rotation of ITBLN, the front view of interface between upper and lower layers shows the defined coordinate angular bisector systems which used for derivation of symmetry of distributions of stress fields.

Using the angular bisector defined coordinate system (see Supplementary Figure 1), the component of particle velocity polarization and corresponding stress can be expressed by amplitudes of partial waves discussed in ***Supplementary II.1***, given as:

$$u_Z^{upper} = (A_\alpha + B_\alpha)\cos\theta_\alpha + (A_\beta + B_\beta)\cos\theta_\beta \qquad \text{(S29)}$$

$$u_Z^{lower} = (C_\alpha + D_\alpha)\cos\theta_\alpha + (C_\beta + D_\beta)\cos\theta_\beta \qquad \text{(S30)}$$

$$u_Y^{upper} = (A_\alpha + B_\alpha)\sin\theta_\alpha - (A_\beta + B_\beta)\sin\theta_\beta \qquad \text{(S31)}$$

$$u_Y^{lower} = -(C_\alpha + D_\alpha)\sin\theta_\alpha + (C_\beta + D_\beta)\sin\theta_\beta \qquad \text{(S32)}$$

$$T_Z^{upper} = (B_\alpha - A_\alpha)Z_\alpha\cos\theta_\alpha + (B_\beta - A_\beta)Z_\beta\cos\theta_\beta \qquad \text{(S33)}$$

$$T_Z^{lower} = (C_\alpha - D_\alpha)Z_\alpha\cos\theta_\alpha + (C_\beta - D_\beta)Z_\beta\cos\theta_\beta \qquad \text{(S34)}$$

$$T_Y^{upper} = (B_\alpha - A_\alpha)Z_\alpha\sin\theta_\alpha + (A_\beta - B_\beta)Z_\beta\sin\theta_\beta \qquad \text{(S35)}$$

$$T_Y^{lower} = (D_\alpha - C_\alpha)Z_\alpha\sin\theta_\alpha + (C_\beta - D_\beta)Z_\beta\sin\theta_\beta \qquad \text{(S36)}$$

where $Z_{\alpha,\beta}$ is acoustic impedance defined as $\rho\omega/k_{\alpha,\beta}$, which can be solved by equation (S17). If the thickness of the two LN layers is the same, marked as h, two series of symmetry can be involved as $A_{\alpha,\beta} = \pm C_{\alpha,\beta}$ as well as $B_{\alpha,\beta} = \pm D_{\alpha,\beta}$. It is evident that the two series exhibite the opposite symmetric of stress and particle velocity distributions as

indicated by equations (S29-S36).

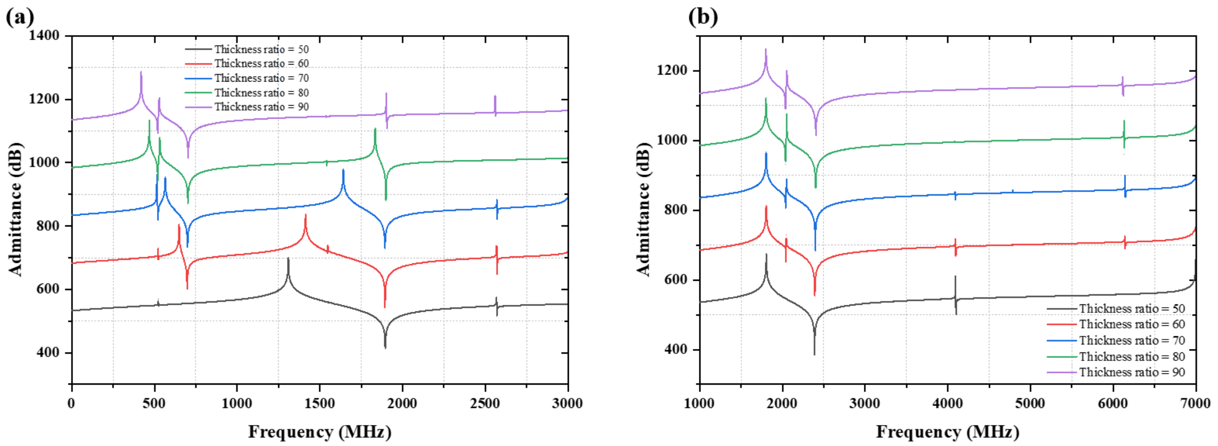

Supplementary Figure 2. Electrical responses of ITBLN at different thickness ratios while the angle were set as **a,** 116° and **b,** 297°.

To be noted that the equations (S29-S36) are valid only on the boundary between the two LN layers if the origin of the X-axis is set at this boundary. As for the distribution characteristics along X direction in the whole cavity, the rotation of polarization would be expected to be remarkable as the result of different phase velocities/wavenumbers of α and β type SH partial waves for superposition of total fields.

Once the thickness of two piezoelectric layers are unequal, the symmetry of fields distribution would be broken. Two extra parasitic modes can be observed, as shown in Supplementary Figure 2. Despite the coupling coefficients of these modes are quite small, the parasitic modes would degrade the performance of acoustic devices in potential applications.

C. Calculation of refraction T and reflection Γ coefficients

In the first case, we consider an α-type polarized SH wave incident from one LN layer into the other LN layer, as seen in Fig.S3(a), at the interface between two layers, there would be two reflected waves(α-type polarized, $\vec{u}_{R\alpha}$ and β-type polarized, $\vec{u}_{R\beta}$) and two refracted waves (α-type polarized, $\vec{u}_{\mathrm{T}\alpha}$ and β-type polarized, $\vec{u}_{\mathrm{T}\beta}$). As a ratio of amplitudes of these waves, the refraction and reflection coefficients (T and Γ) can be involved in the expression of waves at the interface:

$$\vec{u}_{I\alpha} = \hat{y}_1 A e^{-jk_\alpha x} = A(\hat{z}\cos\theta_\alpha + \hat{y}\sin\theta_\alpha)e^{-jk_\alpha x} \tag{S37}$$

$$\vec{u}_{R\alpha} = \hat{y}_1 A\Gamma_{\alpha\alpha} e^{+jk_\alpha x} = A\Gamma_{\alpha\alpha}(\hat{z}\cos\theta_\alpha + \hat{y}\sin\theta_\alpha)e^{+jk_\alpha x} \tag{S38}$$

$$\vec{u}_{R\beta} = \hat{z}_1 A\Gamma_{\beta\alpha} e^{+jk_\beta x} = A\Gamma_{\beta\alpha}(\hat{z}\cos\theta_\beta - \hat{y}\sin\theta_\beta)e^{+jk_\beta x} \tag{S39}$$

$$\vec{u}_{\mathrm{T}\alpha} = \hat{y}_2 A\mathrm{T}_{\alpha\alpha} e^{-jk_\alpha x} = A\mathrm{T}_{\alpha\alpha}(\hat{z}\cos\theta_\alpha - \hat{y}\sin\theta_\alpha)e^{-jk_\alpha x} \tag{S40}$$

$$\vec{u}_{\mathrm{T}\beta} = \hat{z}_2 A\mathrm{T}_{\beta\alpha} e^{-jk_\beta x} = A\mathrm{T}_{\beta\alpha}(\hat{z}\cos\theta_\beta + \hat{y}\sin\theta_\beta)e^{-jk_\beta x} \tag{S41}$$

To simplify the derivation for a more intuitive and concise solutions with physical pictures, the piezoelectricity was neglected here. Thus, the stress of all plane waves presupposed above can be calculated by the combination of

equation (S14) and the following equation:

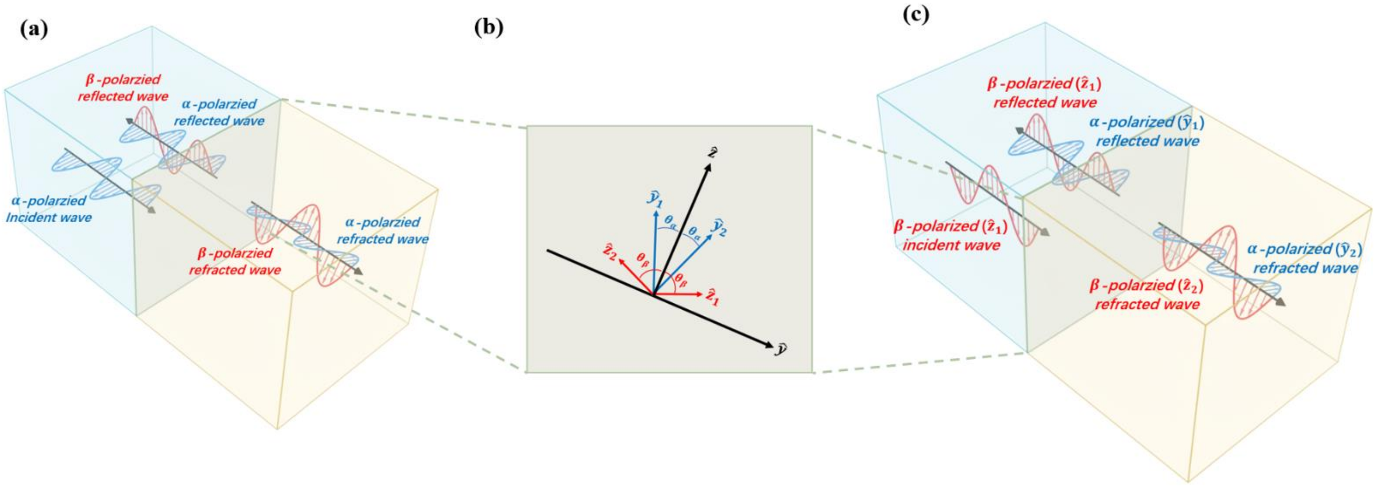

Supplementary Figure 3. Schematic of reflection and refraction of **a**, α-polarized incident waves and **c,** β-polarized incident waves with the **b,** coordinated system used in derivation of coefficients calculations.

$$T_I = c_{IJ} \nabla_{Ji} u_i - e_{Ij} E_j \tag{S42}$$

Before the calculation of refraction and reflection coefficients, the influence of piezoelectricity term $-e_{Ij}E_j$ in equation (S42) on the acoustic impedance needs to be analyzed. Firstly, since either α and β exhibits shear-horizontal polarization (u_y and u_z) and merely couples with longitudinal electric fields (E_x), substituting Eq(S11), (S12) and (S22) into (S42) would lead to nonzero value of T_5 and T_6 as well as the other components remain zero, given as:

$$T_5 = \frac{\partial}{\partial x}\left(c_{56}u_y + c_{55}u_z\right) - e_{15}E_x \tag{S43}$$

$$T_6 = \frac{\partial}{\partial x}\left(c_{66}u_y + c_{56}u_z\right) - e_{16}E_x \tag{S44}$$

The electric field E_x can be calculated by equation (S18). Meanwhile, relations Eq(S2) between strain **S** and displacement $\vec{\boldsymbol{u}}$ gives:

$$S_5 = \frac{\partial u_z}{\partial x} \tag{S45}$$

$$S_6 = \frac{\partial u_y}{\partial x} \tag{S46}$$

Thus, the stress of α and β modes with consideration of piezoelectricity were obtained:

$$T_5 = \frac{\partial}{\partial x}\left[\left(c_{56} + \frac{e_{15}e_{16}}{\epsilon_{11}}\right)u_y + \left(c_{55} + \frac{e_{15}^2}{\epsilon_{11}}\right)u_z\right] \tag{S47}$$

$$T_6 = \frac{\partial}{\partial x}\left[\left(c_{66} + \frac{e_{16}^2}{\epsilon_{11}}\right)u_y + \left(c_{56} + \frac{e_{15}e_{16}}{\epsilon_{11}}\right)u_z\right] \tag{S48}$$

The polarization of SH modes needs to be solved. By dividing with u_z at both sides of the Eq(S17), the dispersion equations can be rewritten as:

$$k^2(c'_{66}\gamma + c'_{56}) = \rho\omega^2\gamma \tag{S49}$$

$$k^2(c'_{56}\gamma + c'_{55}) = \rho\omega^2 \tag{S50}$$

where γ denotes the polarization by $\gamma \equiv u_y/u_z$, and coefficients c'_{66}, c'_{56}, and c'_{55} is defined for simplification as $c_{66} + e_{16}^2/\epsilon_{11}$, $c_{56} + e_{15}e_{16}/\epsilon_{11}$ and $c_{55} + e_{15}^2/\epsilon_{11}$, respectively. Combination of Eq(R49) and (R50) gives a quadratic equation of γ:

$$c'_{56}\gamma^2 + (c'_{55} - c'_{66})\gamma - c'_{56} = 0 \tag{S51}$$

Eq(R51) can be also written as:

$$\gamma(c'_{56}\gamma + c'_{55}) = c'_{66}\gamma + c'_{56} \tag{S52}$$

Here we marked the two solutions to Eq(R51) or (R52) as α and β, which corresponds to the α and β modes solved by Eq(R48). Obviously, both α and β must satisfy the equations above.

Thirdly, with help of the polarization equations derived above, the stress and displacement of α and β modes can be related. Considering a wave propagating alone +x direction with α or β polarization type, its displacement field can be written as:

$$\vec{\boldsymbol{u}} = \left(u_y\hat{y} + u_z\hat{z}\right) \tag{S53}$$

where the harmonic term $\exp(j\omega t - kx)$ is omitted. Substituting γ into Eq(S53) gives:

$$\vec{\boldsymbol{u}} = u_z(\gamma\hat{y} + \hat{z}) \tag{S54}$$

Meanwhile, according to the projection of stress along x axis:

$$\mathbf{T} \cdot \hat{x} = T_1\hat{x} + T_6\hat{y} + T_5\hat{z} \tag{S55}$$

Combination with Eq(S47) and (S48), the projection of stress field can be written as:

$$\mathbf{T} \cdot \hat{x} = -jk\left[\left(c'_{66}u_y + c'_{56}u_z\right)\hat{y} + \left(c'_{56}u_y + c'_{55}u_z\right)\hat{z}\right] \tag{S56}$$

Substituting γ into Eq(S56) gives:

$$\mathbf{T} \cdot \hat{x} = -jku_z(c'_{56}\gamma + c'_{55})(\gamma\hat{y} + \hat{z}) \tag{S57}$$

where relation Eq(S52) was used during the derivation. To be noted that the complex form of displacement and stress field involves an imaginary part in expressions, which defines the phase. On the other hand, the definition of acoustic impedance Z_γ gives:

$$Z_\gamma = \frac{\rho\omega}{k_\gamma} \tag{S58}$$

where γ equals either to the two solutions (α or β). Therefore, the projection stress of α or β mode can be related with its displacement field:

$$\mathbf{T} \cdot \hat{x} = -j\omega Z_\gamma\vec{\boldsymbol{u}} = -Z_\gamma\vec{\boldsymbol{v}} \tag{S59}$$

Furthermore, if the wave propagates along -x direction, its harmonic term should be $\exp(j\omega t + kx)$. Under this

condition, the relation should be:

$$\mathbf{T}\cdot\hat{x} = Z_\gamma \vec{\boldsymbol{v}} \tag{S60}$$

where γ equals either to the two solutions (α or β). Moreover, the phase velocity (calculated as ω/k) of α and β modes can be derived from Eq(S49) or (S50), which provides an expression of the acoustic impedance in terms of material constants:

$$Z_\gamma = \sqrt{\rho(c'_{56}\gamma + c'_{55})} = \sqrt{\rho\gamma^{-1}(c'_{66}\gamma + c'_{56})} \tag{S61}$$

The stress of α-type polarized and β-type polarized waves can be expressed as its acoustic impedance form:

$$(T\cdot\hat{x})_{I\alpha} = -j\omega\hat{y}_1 Z_\alpha A = j\omega Z_\alpha A(-\hat{z}\cos\theta_\alpha - \hat{y}\sin\theta_\alpha) \tag{S62}$$

$$(T\cdot\hat{x})_{R\alpha} = +j\omega\hat{y}_1 Z_\alpha A\Gamma_{\alpha\alpha} = j\omega Z_\alpha A\Gamma_{\alpha\alpha}(\hat{z}\cos\theta_\alpha + \hat{y}\sin\theta_\alpha) \tag{S63}$$

$$(T\cdot\hat{x})_{R\beta} = +j\omega\hat{z}_1 Z_\beta A\Gamma_{\beta\alpha} = j\omega Z_\beta A\Gamma_{\beta\alpha}(\hat{z}\cos\theta_\beta - \hat{y}\sin\theta_\beta) \tag{S64}$$

$$(T\cdot\hat{x})_{\mathrm{T}\alpha} = -j\omega\hat{y}_2 Z_\alpha A\mathrm{T}_{\alpha\alpha} = j\omega Z_\alpha A\mathrm{T}_{\alpha\alpha}(-\hat{z}\cos\theta_\alpha + \hat{y}\sin\theta_\alpha) \tag{S65}$$

$$(T\cdot\hat{x})_{\mathrm{T}\beta} = -j\omega\hat{z}_2 Z_\beta A\mathrm{T}_{\beta\alpha} = j\omega Z_\beta A\mathrm{T}_{\beta\alpha}(-\hat{z}\cos\theta_\beta - \hat{y}\sin\theta_\beta) \tag{S66}$$

Where the $e^{\pm jk_{\alpha,\beta}x}$ was omitted since we take zero point of x-direction at the boundary. Only the boundary continue conditions of elastic fields, equation (S15-S16) shall be considered, leading the coefficients to be solved:

$$\begin{cases} \cos\theta_\alpha + \Gamma_{\alpha\alpha}\cos\theta_\alpha + \Gamma_{\beta\alpha}\cos\theta_\beta = \mathrm{T}_{\alpha\alpha}\cos\theta_\alpha + \mathrm{T}_{\beta\alpha}\cos\theta_\beta \\ \sin\theta_\alpha + \Gamma_{\alpha\alpha}\sin\theta_\alpha - \Gamma_{\beta\alpha}\sin\theta_\beta = \mathrm{T}_{\alpha\alpha}\sin\theta_\alpha - \mathrm{T}_{\beta\alpha}\sin\theta_\beta \\ -Z_\alpha\cos\theta_\alpha + Z_\alpha\Gamma_{\alpha\alpha}\cos\theta_\alpha + Z_\beta\Gamma_{\beta\alpha}\cos\theta_\beta = -Z_\alpha\mathrm{T}_{\alpha\alpha}\cos\theta_\alpha - Z_\beta\mathrm{T}_{\beta\alpha}\cos\theta_\beta \\ Z_\alpha\sin\theta_\alpha - Z_\alpha\Gamma_{\alpha\alpha}\sin\theta_\alpha + Z_\beta\Gamma_{\beta\alpha}\sin\theta_\beta = -Z_\alpha\mathrm{T}_{\alpha\alpha}\sin\theta_\alpha - Z_\beta\mathrm{T}_{\beta\alpha}\sin\theta_\beta \end{cases} \tag{S67 - S70}$$

Secondly, following the same approach, the case as shown in Supplementary Figure 3b that an β-type polarized SH wave incident from one LN layer into the other LN layer would be expressed as:

$$\vec{u}_{I\beta} = \hat{z}_1 Ae^{-jk_\beta x} = A(\hat{z}\cos\theta_\beta - \hat{y}\sin\theta_\beta)e^{-jk_\beta x} \tag{S71}$$

$$\vec{u}_{R\alpha} = \hat{y}_1 A\Gamma_{\alpha\beta}e^{+jk_\alpha x} = A\Gamma_{\alpha\beta}(\hat{z}\cos\theta_\alpha + \hat{y}\sin\theta_\alpha)e^{+jk_\alpha x} \tag{S72}$$

$$\vec{u}_{R\beta} = \hat{z}_1 A\Gamma_{\beta\beta}e^{+jk_\beta x} = A\Gamma_{\beta\beta}(\hat{z}\cos\theta_\beta - \hat{y}\sin\theta_\beta)e^{+jk_\beta x} \tag{S73}$$

$$\vec{u}_{\mathrm{T}\alpha} = \hat{y}_2 A\mathrm{T}_{\alpha\beta}e^{-jk_\alpha x} = A\mathrm{T}_{\alpha\beta}(\hat{z}\cos\theta_\alpha - \hat{y}\sin\theta_\alpha)e^{-jk_\alpha x} \tag{S74}$$

$$\vec{u}_{\mathrm{T}\beta} = \hat{z}_2 A\mathrm{T}_{\beta\beta}e^{-jk_\beta x} = A\mathrm{T}_{\beta\beta}(\hat{z}\cos\theta_\beta + \hat{y}\sin\theta_\beta)e^{-jk_\beta x} \tag{S75}$$

and its stress fields can be calculated as

$$(T\cdot\hat{x})_{I\beta} = -j\omega\hat{z}_1 Z_\beta A = j\omega Z_\beta A(-\hat{z}\cos\theta_\beta + \hat{y}\sin\theta_\beta) \tag{S76}$$

$$(T\cdot\hat{x})_{R\alpha} = +j\omega\hat{y}_1 Z_\alpha A\Gamma_{\alpha\beta} = j\omega Z_\alpha A\Gamma_{\alpha\beta}(+\hat{z}\cos\theta_\alpha + \hat{y}\sin\theta_\alpha) \tag{S77}$$

$$(T\cdot\hat{x})_{R\beta} = +j\omega\hat{z}_1 Z_\beta A\Gamma_{\beta\beta} = j\omega Z_\beta A\Gamma_{\beta\beta}(+\hat{z}\cos\theta_\beta - \hat{y}\sin\theta_\beta) \tag{S78}$$

$$(T \cdot \hat{x})_{\mathrm{T}\alpha} = -j\omega\hat{y}_2 Z_\alpha A\mathrm{T}_{\alpha\beta} = j\omega Z_\alpha A\mathrm{T}_{\alpha\beta}(-\hat{z}\cos\theta_\alpha + \hat{y}\sin\theta_\alpha) \tag{S79}$$

$$(T \cdot \hat{x})_{\mathrm{T}\beta} = -j\widehat{\omega z}_2 Z_\beta A\mathrm{T}_{\beta\beta} = j\omega Z_\beta A\mathrm{T}_{\beta\beta}(-\hat{z}\cos\theta_\beta - \hat{y}\sin\theta_\beta) \tag{S80}$$

The resulting equations for solving coefficients are:

$$\begin{cases} \cos\theta_\beta + \Gamma_{\alpha\beta}\cos\theta_\alpha + \Gamma_{\beta\beta}\cos\theta_\beta = \mathrm{T}_{\alpha\beta}\cos\theta_\alpha + \mathrm{T}_{\beta\beta}\cos\theta_\beta \\ -\sin\theta_\beta + \Gamma_{\alpha\beta}\sin\theta_\alpha - \Gamma_{\beta\beta}\sin\theta_\beta = -\mathrm{T}_{\alpha\beta}\sin\theta_\alpha + \mathrm{T}_{\beta\beta}\sin\theta_\beta \\ -Z_\beta\cos\theta_\beta + Z_\alpha\Gamma_{\alpha\beta}\cos\theta_\alpha + Z_\beta\Gamma_{\beta\beta}\cos\theta_\beta = -Z_\alpha\mathrm{T}_{\alpha\beta}\cos\theta_\alpha - Z_\beta\mathrm{T}_{\beta\beta}\cos\theta_\beta \\ Z_\beta\sin\theta_\beta + Z_\alpha\Gamma_{\alpha\beta}\sin\theta_\alpha - Z_\beta\Gamma_{\beta\beta}\sin\theta_\beta = Z_\alpha\mathrm{T}_{\alpha\beta}\sin\theta_\alpha - Z_\beta\mathrm{T}_{\beta\beta}\sin\theta_\beta \end{cases} \tag{S81 - S84}$$

When the thickness of the two LN layers are the same, in which the symmetry and anti-symmetry of modes in IBLN resonant cavity involving as $A_{\alpha,\beta} = \pm C_{\alpha,\beta}$ and $B_{\alpha,\beta} = \pm D_{\alpha,\beta}$ can be used into simplify of equations derived above. Consequently, the dispersion of two series of eigenmodes have an intuitional formalism:

$$-\frac{\tan(F)}{\tan(rF)} = \begin{cases} r\tan^2(\theta - \theta_{\beta 0}), for\ symmetric\ T_Z\ and\ antisymmetric\ T_Y \\ r^{-1}\tan^2(\theta_{\beta 0} - \theta), for\ symmetric\ T_Y\ and\ antisymmetric\ T_Z \end{cases} \tag{S85}$$

where θ is half-twisted angle, $\theta_{\beta 0}$ is original polarization angle of $r = Z_\alpha / Z_\beta$ and $F = \mathrm{h}\omega / V_\alpha$, and the relation of $\theta_\alpha + \theta_\beta = \pi/2$ has been used in derivation (see Supplementary Figure 4).

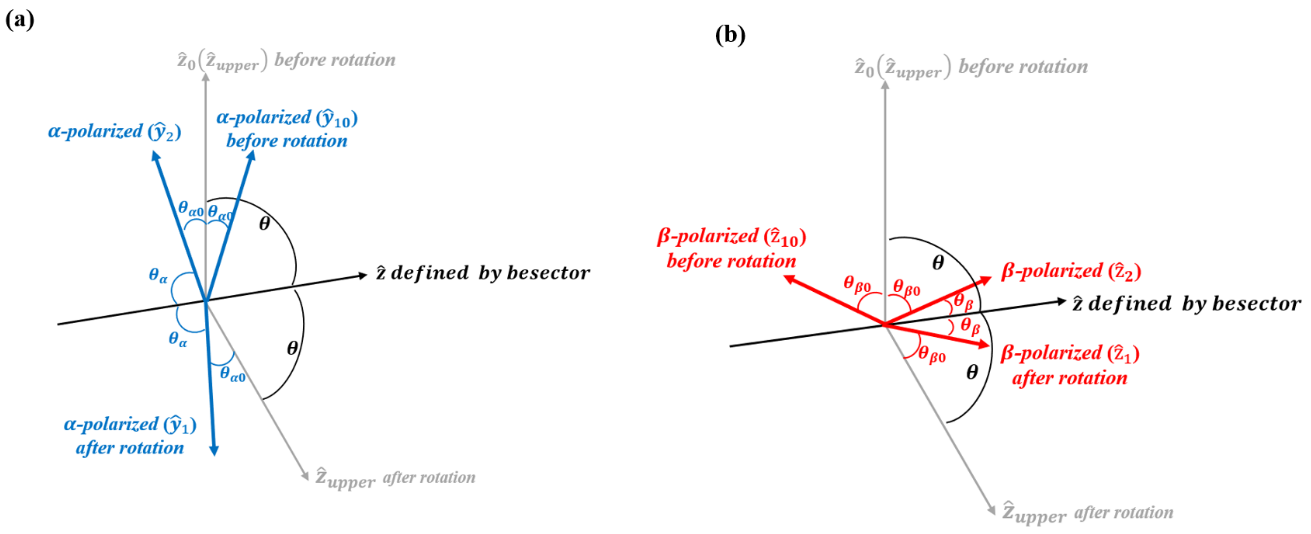

Supplementary Figure 4. Schematic of relations of half-rotation angle θ and polarization angle. The defined angles of polarizations agree with defined angles in Supplementary Figure 3b. **a,** Relations of θ and α-polarization. **b,** Relations of θ and β-polarization.

D. Calculation of the electromechanical coupling coefficient K^2

The definition of electromechanical coupling coefficient K^2 was indicated in equation (3) in Section III, in which the molecule called as the mutual energy U_m was calculated as:

$$U_m = \frac{1}{4}\int \left(T\colon d \cdot \vec{E} + \vec{E} \cdot d\colon T\right) dV \tag{S86}$$

where the integrand can be expressed in Voigt notation:

$$T\colon d \cdot \vec{E} + \vec{E} \cdot d\colon T = T_I d_{Ij} E_j + E_i d_{iJ} T_J \tag{S87}$$

As for piezoelectrically stiffened orthogonal shear horizontal polarized modes in ITBLN resonant cavity, only the shear horizontal stress T_5 and T_6 and longitudinal electric field E_x exist, according to equation (S13-S14). Thus, the integral can be calculated as following components form:

$$T\colon d \cdot \vec{E} + \vec{E} \cdot d\colon T = T_5 d_{51} E_x + T_6 d_{61} E_x + E_x d_{15} T_5 + E_x d_{16} T_6 = 2(T_5 d_{15} + T_6 d_{16}) E_x \tag{S88}$$

where the Hermitian symmetry of piezoelectric strain constants was used. Through the same derivations, the integrand of elastic energy U_e and electric energy U_d can be calculated as:

$$T\colon s^E\colon T = T_I s_{IJ} T_J = s_{55} T_5^2 + s_{66} T_6^2 + 2 s_{56} T_5 T_6 \tag{S89}$$

$$\vec{E} \cdot \varepsilon^S \cdot \vec{E} = E_i \varepsilon_{ij} E_j = \varepsilon_{11} E_x^2 \tag{S90}$$

Next, to introduce the symmetry of distribution of stress of solved eigenmodes in ITBLN resonant cavity, the corresponding shear horizontal stress T_5 and T_6 should be re-written in the coordinate angular bisectors systems (see Fig.S3) expressed respectively in upper and lower LN layers as:

$$T_5^{up} = T_Z \cos\theta + T_Y \sin\theta \tag{S91}$$

$$T_6^{up} = T_Z \sin\theta - T_Y \cos\theta \tag{S92}$$

$$T_5^{down} = T_Z \cos\theta - T_Y \sin\theta \tag{S93}$$

$$T_6^{down} = T_Z \sin\theta + T_Y \cos\theta \tag{S94}$$

Substituting the four equations above into equation (S31) then the mutual energy of shear horizontal polarized eigenmodes in ITBLN can be calculated as:

$$\begin{aligned} U_m = &\frac{1}{2}\int_{V^{up}+V^{down}} E_x T_Z (d_{15}\cos\theta + d_{16}\sin\theta) dV \\ &+ \frac{1}{2}\int_{V^{up}} E_x T_Y (d_{15}\sin\theta - d_{16}\cos\theta) dV \\ &+ \frac{1}{2}\int_{V^{down}} E_x T_Y (-d_{15}\sin\theta + d_{16}\cos\theta) dV \end{aligned} \tag{S95}$$

which is the question (5) in manuscript.

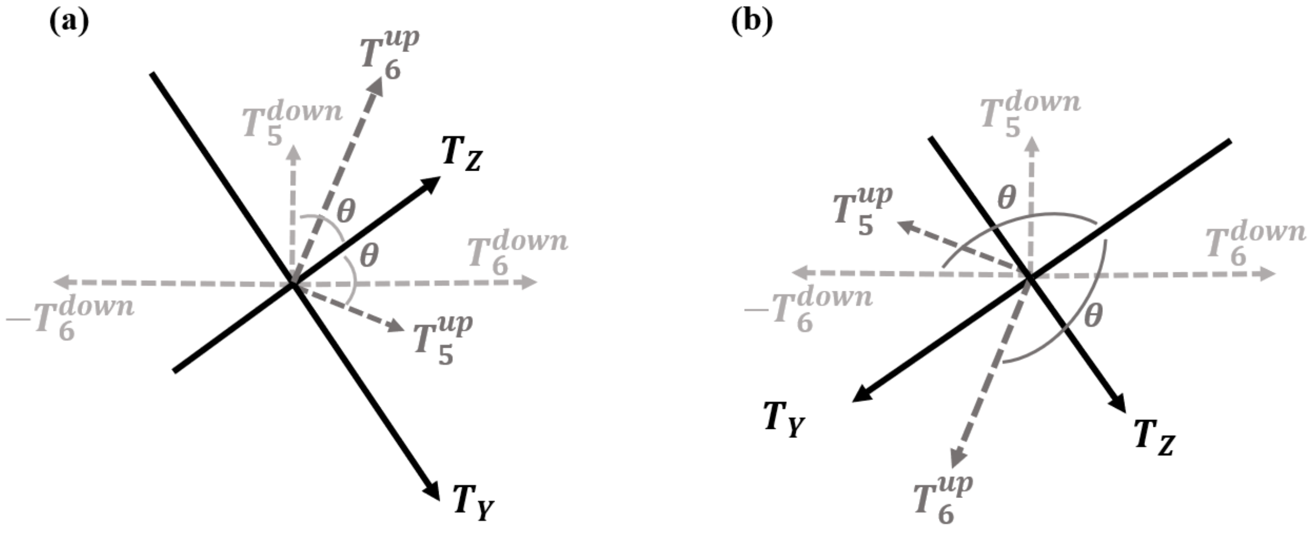

Supplementary Figure 5. Schematics of coordinate angular bisector systems at **a,** 116° and **b,** 296°

Supplementary Note 3. Shear horizontal modes in single layer LN resonant cavity

Supplementary Figure 6 shows the FEM simulating results of single LN layer with same crystal orientation and exciting electric field as a contrast. The resonant modes are distinguished into series of α- type SH standing waves and series of β- type SH standing waves, and only symmetric modes excited while antisymmetric modes disappear (See the left part of the upper row of Supplementary Figure 6, the dashed line indicates that these modes would not be excited owing to the antisymmetric distributions of stress), which means these two orthogonally polarized SH waves would degenerate because of lack of the boundary between two different crystal orientation LN layers as exhibited in ITBLN.

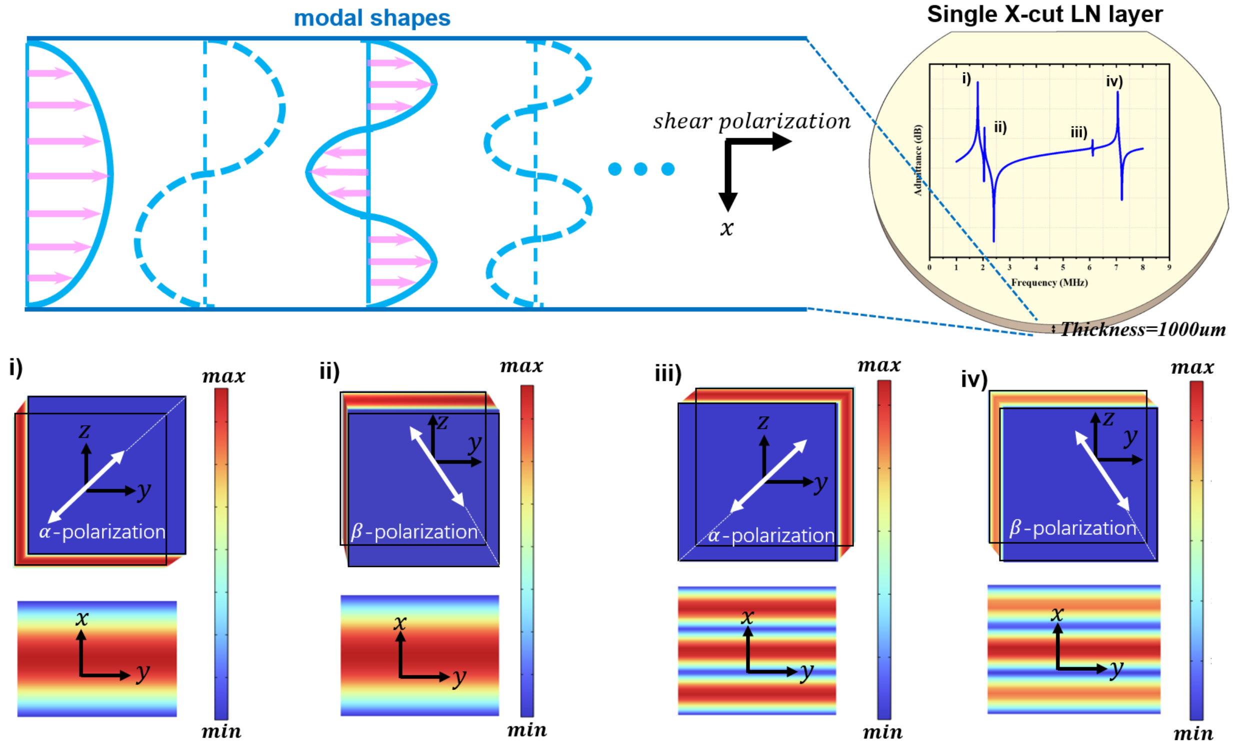

Supplementary Figure 6. upper row, FEM-simulated admittance curves of single *X*-cut LN layers and its modes profiles for shear-horizontal standing waves, lower row, polarization types and stress distributions of first four order shear-horizontal modes, the white arrows indicate the polarization directions.

The right part of the upper row of Supplementary Figure 6 shows the simulated admittance of single X-cut LN layers. The existing four peaks correspond to the first and second order of α and β type SH standing waves, respectively. Their polarization were verified by the relative dislocation between the bottom and top surfaces, as shown in the lower row of Supplementary Figure 6, marked as white arrows. The orders were determined by distributions of shear stress in the cross section, which were also plotted in Supplementary Figure 6.

Supplementary Note 4. FEM simulation of ITBLN resonant cavity

Our FEM simulation was preformed based on the COMSOL Multiphysics. Two layers of $LiNbO_3$ with size of 2um × 2um wide and 500nm thick were stacked. At the top surface of the upper $LiNbO_3$ layer and bottom surface of the lower $LiNbO_3$ layer, two electric terminals with equivalent electrical potential (1V and 0V respectively) were added to simulate the top and bottom electrodes and excite acoustic resonate. Two pairs of periodic boundaries, more precisely as continuous conditions, were added on both sides of layers. Since the structure of twisted bilayer $LiNbO_3$ requires the head-to-head set of X-axis between the upper and lower layers and exhibits a twisting angle (θ_T), we utilized the Euler angles (α, β, γ) systems in both layers, making ($-\theta_T$, $-90°$, 90°) for the upper layer and (0°, $-90°$, $-90°$) for the lower layer. We employ two simulation modules: solid mechanics and electrostatics while the strain and electrostatic fields are coupled together via the piezoelectric effects.

The materials properties of $LiNbO_3$ we used in our paper are listed as follows:

Supplementary Table 1. Materials properties of $LiNbO_3$

Elastic constants	Values ($10^9\ N/m^2$)
c_{11}^E	203
c_{12}^E	53.0
c_{13}^E	75.0
c_{14}^E	9.0
c_{33}^E	245
c_{44}^E	60.0
Piezoelectric stress constants	Values (C/m^2)
e_{15}	3.7
e_{22}	2.5
e_{31}	0.2
e_{33}	1.3
Permittivity	Values ($10^{-9}F/m$)
ε_{11}	0.390
ε_{33}	0.257

Parameters are cited from: Warner, A.W.; Onoe, M.; Coquin, G.A. Determination of Elastic and Piezoelectric Constants for Crystals in Class (3m). J. Acoust. Soc. Am. 1967, 42, 1223–1231.

Supplementary Note 5. Details of the coordinate angular bisectors systems and corresponding shear horizontal stress

In this section, we give a more detailed description and definitions of coordinate angular bisectors systems and explain the relationship between the systems and corresponding shear horizontal stress, as a complement of derivation in former sections of this supplementary materials.

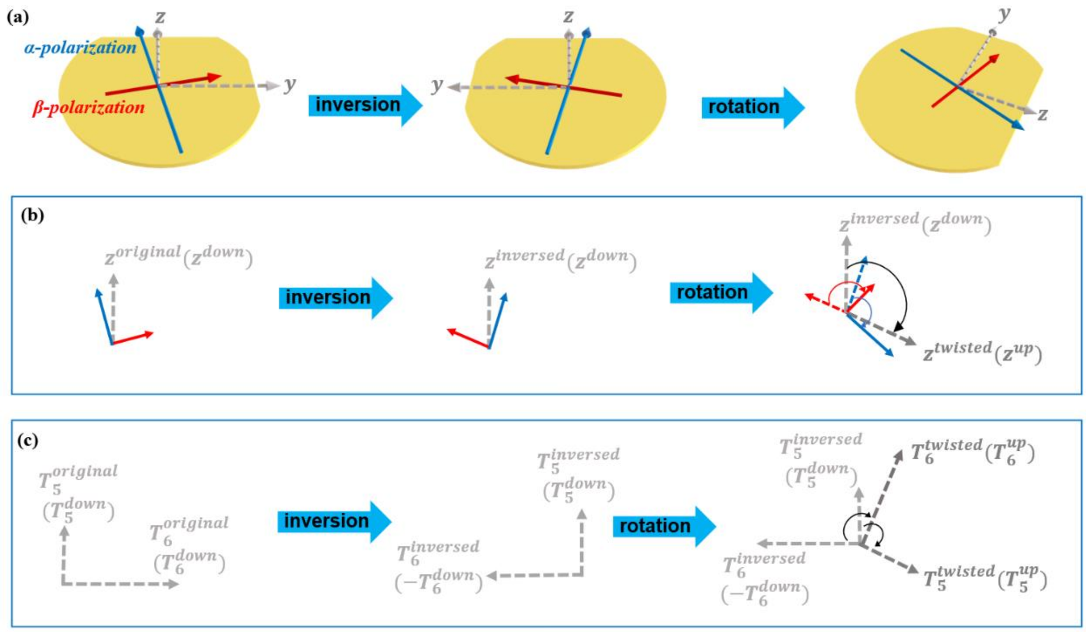

Supplementary Figure 7. Schematic of **a,** the original single layer of X-cut $LiNbO_3$, **b,** the inversed layer of X-cut $LiNbO_3$ and **c,** the layer of X-cut $LiNbO_3$ after the inversion and rotation, the dashed arrows in the bottom row indicate the corresponding shear horizontal stress.

First of all, the directions of polarizations and shear stress of α and β modes in the upper and lower LN layers are different, since one of layers experienced an inversion and rotation, as shown in Supplementary Figure 7a. Consistent with Supplementary Figure 1, the upper layer (marked as "up" in the corresponding superscripts) experienced an inversion along z axis and then twisted with a rotation angle (2θ), while the lower layer (marked as "down" in the corresponding superscripts) remains unchanged as the original layer. The original directions of α and β shear-horizontal polarization and their changes after the inversion (marked as "inversed" in the corresponding superscripts) and rotation (marked as "twisted" in the corresponding superscripts) was schematically extracted form Supplementary Figure 7a, and singly shown in Supplementary Figure 7b with blue arrow indicates α polarization as well as red arrow indicates the β. Meanwhile, axis of y and z can also be treated as directions of T_6 and T_5. As shown in Supplementary Figure 7c, the dashed arrows in light gray on the $LiNbO_3$ films indicate the y and z axis, which can also be marked as $T_6^{original}$ (or T_6^{down}) and $T_5^{original}$ (or T_5^{down}), respectively. After the inversion along z axis, the polarization and y axis of the layer was marked as $T_5^{inversed}$ (which is same as $T_5^{original}$) and $T_6^{inversed}$ (which is the opposite of $T_6^{original}$). Lastly, the dashed arrows in dark gray indicate the stress components $T_6^{twisted}$ (or T_6^{up}) along y axis and $T_5^{twisted}$ (or T_5^{up}) along z axis after the rotation from $T_6^{inversed}$ and $T_5^{inversed}$, respectively.

In Supplementary Figure 3, the coordinate bisectors system was defined as the angular bisectors between the polarizations of same mode in upper and lower layers. Here we provided a more detailed schematics. As shown in Supplementary Figure 8, the arrows indicating the z axis before and after the inversion and rotation and the corresponding polarizations of α and β modes in the rightmost of Supplementary Figure 7b was decomposed into two series: z axis and α polarization as well as z axis and β polarization. From both series, the angular bisector (marked as Z) of $z^{original}$ (or z^{down}) and $z^{twisted}$ (or z^{up}) is also be the angular bisector of original α (or β) polarization and twisted α (or β) polarization. Therefore, the coordinate angular bisectors system can be simply obtained with the α and β polarizations in upper (after inversion and rotation) and lower layers, which denotes the Supplementary Figure 3.

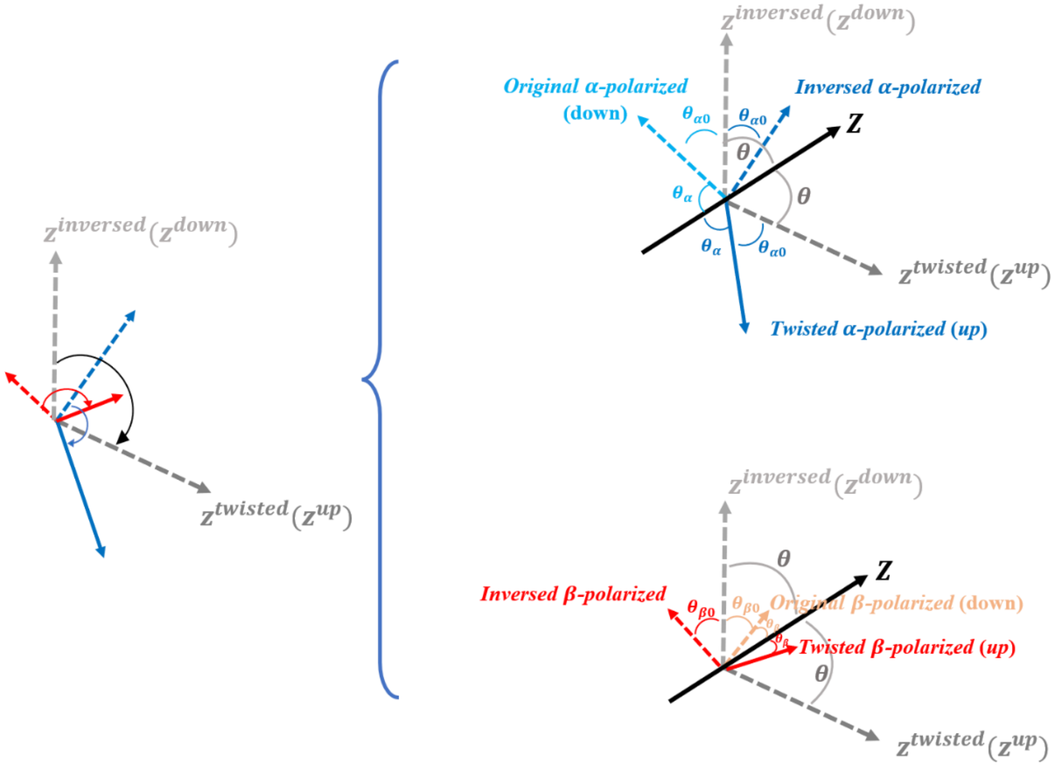

Supplementary Figure 8. Schematic of the definition of the shear horizontal stress components T_Y and T_Z in the coordinate angular bisectors systems at 116°.

Based on the discussion above, here we have defined the shear horizontal stress components T_Y and T_Z in the coordinate angular bisectors systems. As shown in Supplementary Figure 9, the direction of T_Z is defined as the angular bisector between the $T_5^{inversed}$ (or T_5^{down}) and the $T_5^{twisted}$ (or T_5^{up}), as well as T_Y defined as the angular bisector between the $T_6^{inversed}$ (or T_6^{down}) and the $T_6^{twisted}$ (or T_6^{up}). The schematic of the relationship between the corresponding shear horizontal stress (T_5, T_6) and T_Z, T_Y with the twisting angle (2θ) equaling to 296° is also shown in Supplementary Figure 10. To be noted that the defined bisectors systems T_Y and T_Z must exhibit the same chirality in the 116° rotation and 296° rotation, guarantying that equations from (S91) to (S94) being validated.

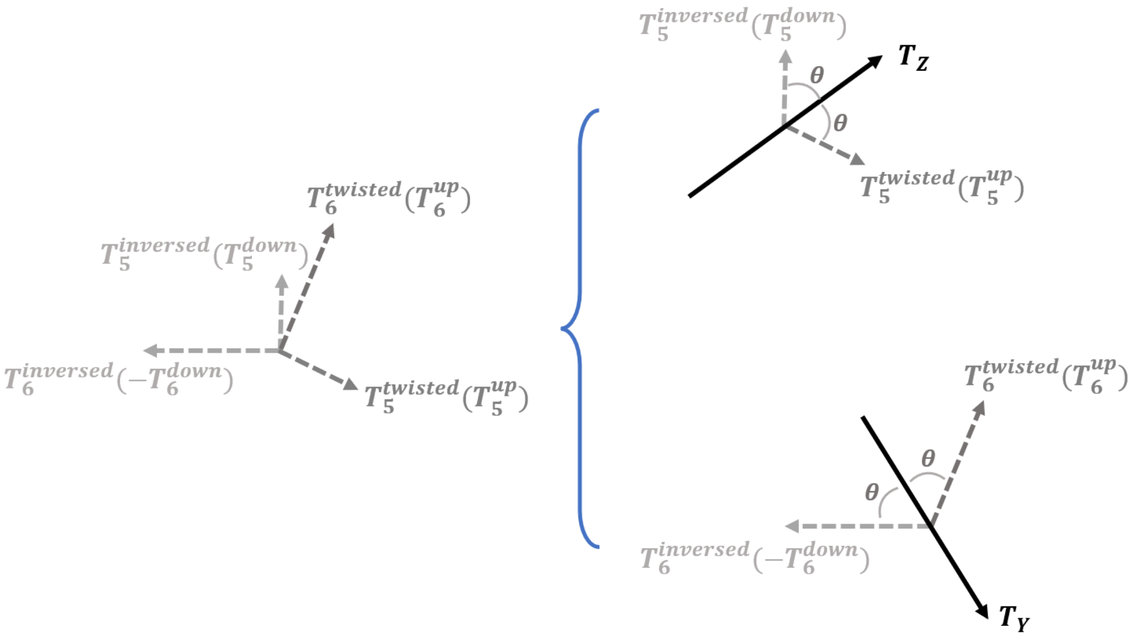

Supplementary Figure 9. Schematic of the definition of the shear horizontal stress components T_Y and T_Z in the coordinate angular bisectors systems at 116°.

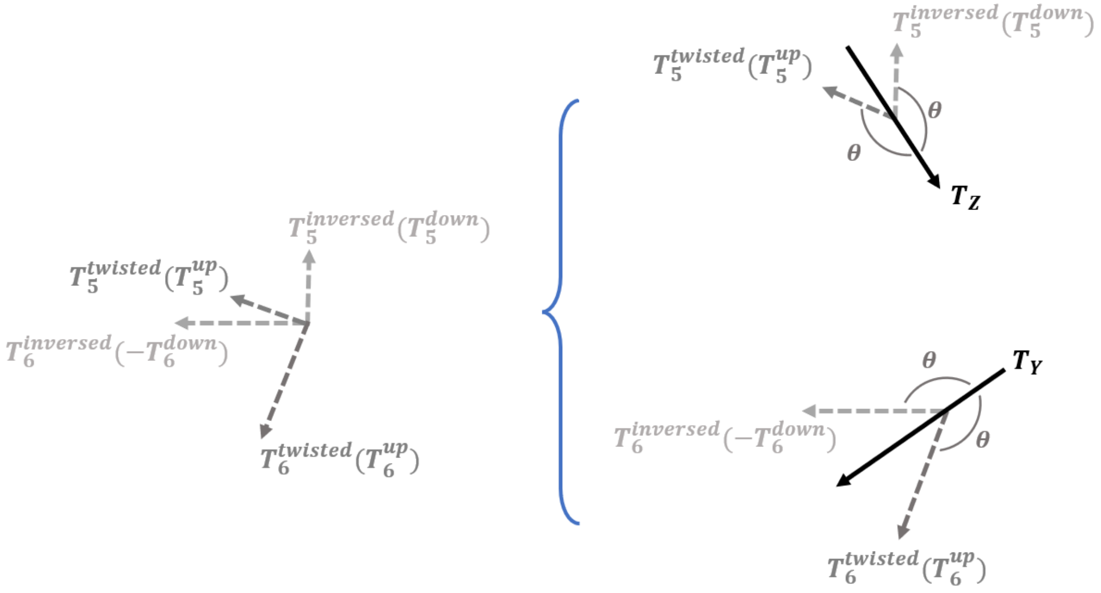

Supplementary Figure 10. Schematic of the definition of the shear horizontal stress components T_Y and T_Z in the coordinate angular bisectors systems at 296°.

Supplementary Note 6. Experimental setup for measurement of ITBLN acoustic resonators.

The schematic of the measurement system is illustrated in Supplementary Figure 11a, which consists of three parts: the single/bi-layer piezoelectric sample with double-sided electrodes, printed circuit board (PCB) and the vector network analyzer (VNA). The PCB and VNA are connected through the RF coaxial cable, and the sample and the PCB are connected through the bonding wires. The actual experiment set up photo is presented in Supplementary Figure 11b. The S_{11} response can be directly converted into admittance (Y), and shown in the screen of the VNA. The zoom-in photos (Supplementary Figure 11c and d) show the details of the PCB and bonding wires in the front-side.

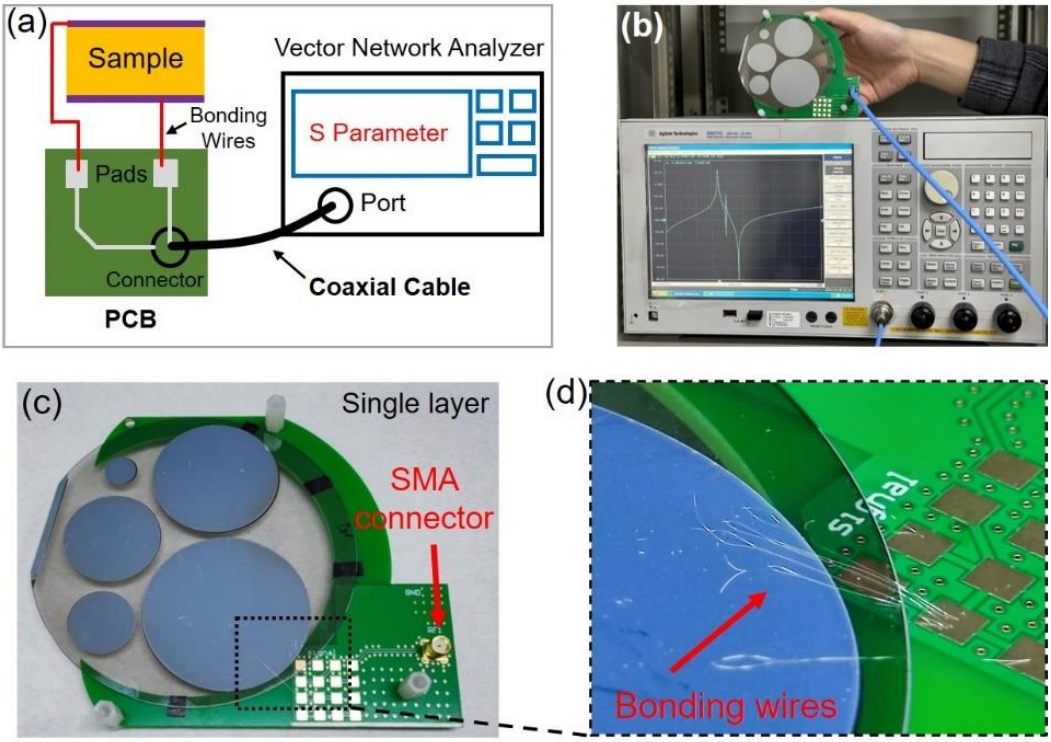

Supplementary Figure 11. Schematic and photos of experimental setup for measurement. **a,** Schematic of the measurement system. Detailed photos of **b,** measurement system, **c,** printed circuit board and **d,** bonding wires.

Supplementary Figure 12a characterized the interface of the bonded bilayer $LiNbO_3$ using cross-sectional transmission electron microscope (TEM) and energy disperse spectroscopy (EDS), and the results are shown below. The interface between the upper and lower $LiNbO_3$ plates is clearly visible, showing a direct material transition without any intermediate or buffer layers. The atomic arrangement of the lower layer is clear due to the formation of strong electron interference. In contrast, the image of the atomic arrangement of the lower layer is blurred because of the twisted angle in the bilayer structure. In other words, it is difficult to see the atomic arrangement of different crystal orientations at the same time. Supplementary Figure 11b and c show the distribution of Nb and O elements, respectively, confirming that both the upper and lower materials are $LiNbO_3$. Therefore, the partial waves in the two layers satisfy the continuous boundary condition at the interface, and the symmetry properties induced by twisting is retained.

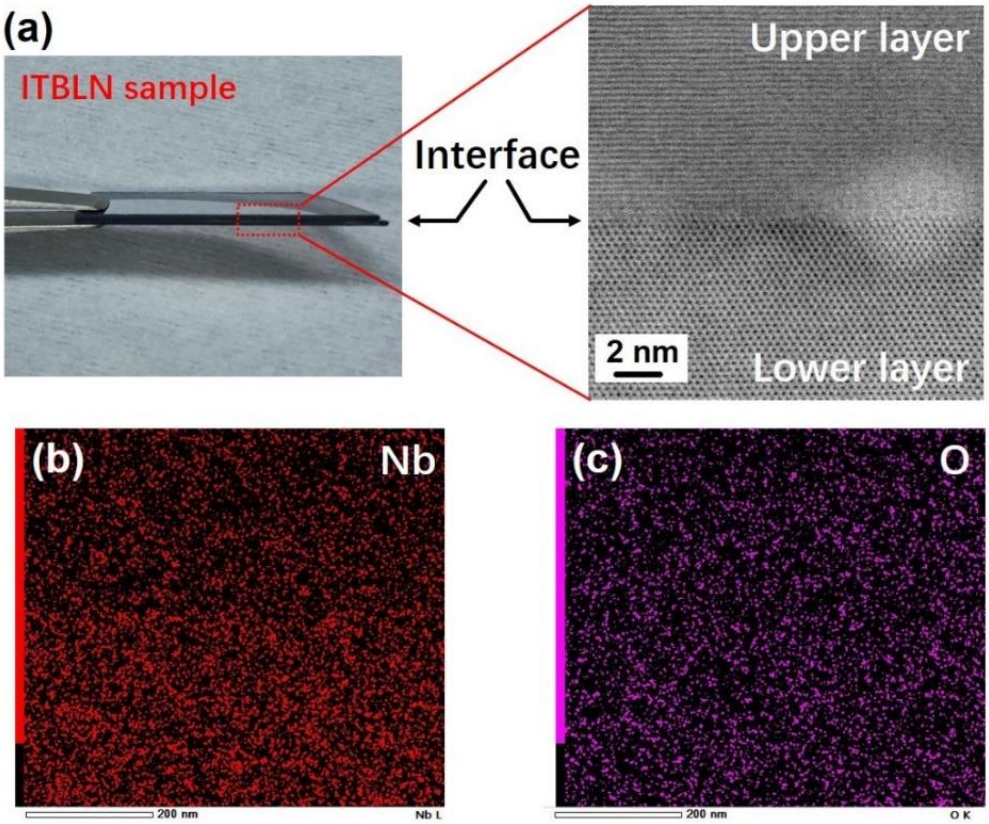

Supplementary Figure 12. Characterization of the interface of the bonded bilayer $LiNbO_3$ **a,** TEM image of the interface of bilayer LiNbO3 plate. **b,** Nb and **c,** O element distribution at the interface region.
